# MVP Expression Facilitates Tumor Cell Proliferation and Migration Supporting the Metastasis of Colorectal Cancer Cells

**DOI:** 10.3390/ijms222212121

**Published:** 2021-11-09

**Authors:** Paulina Pietras, Marta Leśniczak-Staszak, Aldona Kasprzak, Małgorzata Andrzejewska, Karol Jopek, Mateusz Sowiński, Marcin Rucinski, Shawn M. Lyons, Pavel Ivanov, Witold Szaflarski

**Affiliations:** 1 Department of Histology and Embryology, Poznan University of Medical Sciences, 61-701 Poznań, Poland; paulinapietras29@gmail.com (P.P.); marta.m.lesniczak@gmail.com (M.L.-S.); akasprza@ump.edu.pl (A.K.); mandrzej@ump.edu.pl (M.A.); karoljopek@ump.edu.pl (K.J.); matsow@gmail.com (M.S.); marcinruc@ump.edu.pl (M.R.); 2 Department of Biochemistry, Boston University School of Medicine, Boston, MA 02118, USA; smlyons1@bu.edu; 3 The Genome Science Institute, Boston University School of Medicine, Boston, MA 02118, USA; 4 Division of Rheumatology, Immunology, and Allergy, Brigham and Women’s Hospital, Boston, MA 02115, USA; 5 Harvard Medical School, Boston, MA 02115, USA; 6 The Broad Institute of Harvard and M.I.T., Cambridge, MA 02142, USA

**Keywords:** MVP, TEP1, vPARP, vaults, colorectal cancer, metastasis

## Abstract

Cancer cells show significant dysregulation of genes expression, which may favor their survival in the tumor environment. In this study, the cellular vault’s components MVP (major vault protein), TEP1 (telomerase-associated protein 1) and vPARP (vault poly(ADP-ribose) polymerase) were transiently or completely inhibited in U2OS cells (human bone osteosarcoma epithelial cells) to evaluate their impact on the cell proliferative and migratory capacity as well as on the development of their resistance to the drug vinorelbine. Comparative analysis of MVP protein expression level in normal colon tissue, primary colorectal tumor, and metastasis showed that the expression of this protein does not increase significantly in the primary tumor, but its expression increases in metastatic cells. Further comparative molecular analysis using the whole transcriptome microarrays for MVP-positive and MVP-negative cells showed that MVP is involved in regulating proliferation and migration of cancer cells. MVP may facilitate metastasis of colon cancer due to its impact on cell migration. Moreover, two vault proteins, MVP and TEP1, contribute the resistance to vinorelbine, while vPARP does not.

## 1. Introduction

Cellular vaults are cytoplasmic ribonucleoprotein structures with a still unclear role in the cell, although their participation in the development of drug resistance in cancer cells has already been demonstrated [1,2]. The MVP protein is one of the vaults compounds that was first described in 1986 [3]. In addition to MVP, these structures also contain the proteins TEP1 and vPARP as well as non-coding RNAs—vtRNAs [4]. In 1995, it was shown that MVP has the same sequence as LRP (lung resistance protein), whose expression correlates with the development of drug resistance in cancer cells [5]. It then became clear that most likely whole vaults could be involved in this process. Research conducted over the years has confirmed the role of vaults in multidrug resistance [1]. However, it is not fully clear which vault components is particularly responsible for this resistance. It seems that non-coding RNAs, which may temporarily bind to vaults, may play a significant role [6,7].

Cellular vaults are barrel-like structures. Each individual vault molecule consists of 78 molecules of MVP, which are responsible for the formation of the barrel shape [8]. The barrel is made of two symmetrical halves which are joined together in the central protuberance. Each of the halves is made up of 39 MVP proteins, which together form a 39-fold symmetry. A single vault particle has a height of about 67 nm and a width measured in the middle of about 40 nm. The barrel structure narrows down to the poles, where they form caps with a height of approx. 15.5 nm and a diameter of approx. 20 nm. A barrel wall that is only 2 nm thick limits an internal space of approximately 62 nm in length and approximately 35 nm in diameter and is therefore large enough to accommodate most of the cytoplasmic objects in the cell (e.g., several ribosomes).

The crystal structure of vaults shows MVP [8] exclusively, which was surprising because previous biochemical and structural studies also described the TEP1 and vPARP proteins in vaults [9]. However, it was suspected that the higher electron density observed in the regions of both vault caps may correspond to the location of TEP1 [8]. Moreover, the previously described ratio of MVP to other vault components (i.e., TEP1, vPARP and vRNA) being approximately 1:8 [10] excludes the possibility of all these components forming a molecule with 39-fold symmetry revealed in the crystal structure [8]. This indicates that the TEP1 and vPARP bind only to a certain fraction of vaults.

The discovery mentioned above where it was found that MVP is the same protein as LRP, opened a discussion on the role of the MVP protein and whole vaults in the mechanism of multidrug resistance in different types of cancers (see reviews [1,11,12]). Their natural presence in epithelial tissue perfectly matches their potential role as cytostatic protective agents. Thus, vaults act in cancer cells in a similar way to how they protect a healthy cell. There is presently no doubt that vaults are involved in the acquisition of resistance to anticancer drugs.

Current data indicate the participation of MVP not only in multidrug resistance, but also in the aggressiveness of the tumor cells, as presented for the glioblastoma multiforme model [13]. In this study, the expression of MVP by siRNA was reduced causing the impairment of migratory and invasive competencies and decreasing the resistance of glioblastoma cells to starvation in vitro and in vivo. The higher aggressiveness was based on MVP-mediated stabilization of the epidermal growth factor receptor (EGFR)/phosphatidyl-inositol-3-kinase (PI3K) axis. Consequently, overexpression of MVP resulted in a higher invasion rate of human glioblastoma xenograft models [13]. In our studies, we extended the above research. We performed studies on the clinical material and applied the CRISPR-Cas9 method to obtain genetic knockout of MVP. Moreover, we used the DNA microarray technique to compare whole-genome expression in MVP-positive and MVP-negative cells.

Here, we demonstrated that MVP supports tumor metastasis in colon cancer and the migratory capability of malignant cells. First, we showed on clinical material from patients that the amount of MVP does not increase in the primary tumor as compared to healthy tissue. However, large individual changes in particular patients were observed. In contrast, we reached for the first time an important observation that MVP increases in metastatic cancer cells. This observation opens a new direction of research, showing the new role of MVP, and probably also vaults, in the processes responsible for the formation of metastasis.

In our research, we were able to confirm at the molecular level that MVP is associated with the processes of adhesion and migration. We have shown that MVP-negative cells generated using the CRISPR-Cas9 have a lower migration capability, which may result from the observed modulation of the AKT pathway. Moreover, the analysis of total gene expression in parental and MVP-negative cells indicated disorders in adhesion and proliferation mechanisms in the latter cells. In fact, total inhibition of MVP expression in certain cancer cell types (HAP1) resulted in the death of the cells’ population.

## 2. Results

### 2.1. MVP Is Highly Expressed in Tumor Metastasis

**Figure 1:**
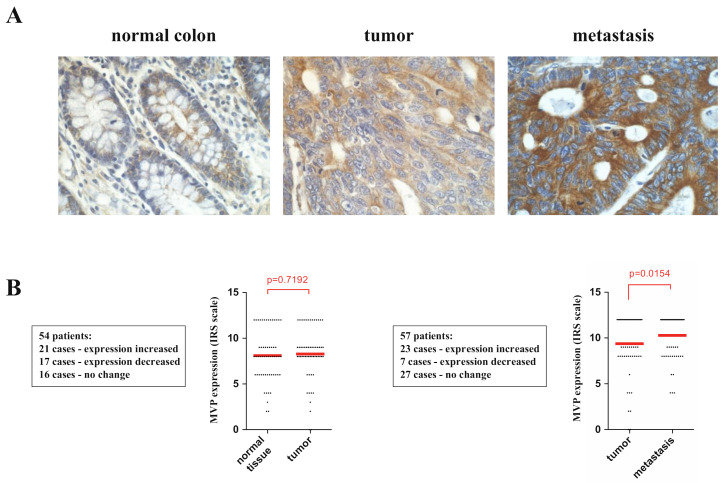
Comparative evaluation of MVP immunohistochemical (IHC) expression in tissue samples of normal colon, colorectal cancer and lymph node metastasis using immunoreactive score (IRS). (**A**) Representative images of the IHC detection of MVP in the normal human colon mucosa, primary tumor and lymph node metastasis in the same patient; note the predominantly cytoplasmic expression pattern of MVP and the most intense IHC reaction in tumor metastasis. Hematoxylin counterstained. 40× objective. (**B**) The comparison of mean MVP expression—left graph: comparison normal tissue—primary tumor and right graph: comparison primary tumor—tumor metastasis (red line–average). The cages show information on the number of patients and information on the increase, decrease and no change in expression.

Tissue samples from 54 patients with colorectal cancer were included for the comparative analysis of the expression in normal tissue vs. primary tumor, and tissue samples from 57 patients to compare expression in primary tumor vs. lymph node metastasis. Detection of immunohistochemical (IHC) reaction for MVP was demonstrated in all tissue types (control, tumor, metastasis). Cytoplasmic MVP expression in the epithelial cells was predominant regardless of the type of tissue tested. The most intense immunoreactivity to MVP was in tissues with metastatic tumor cells (Figure 1A). The expression of the MVP in the control tissue and the primary tumor site shows a high degree of individual variation in relation to individual patients. However, there is no statistically significant increase or decrease in MVP expression in both analyzed groups (*p* = 0.7192) (Figure 1B). In contrast, MVP expression was significantly increased in the group consisting of metastases in lymph nodes. Tissue samples with metastases of 23 patients showed increased expression of MVP, and decreased expression of MVP was observed in seven patients (*p* = 0.0154) (Figure 1B).

### 2.2. Expression of MVP Is Critical to the Survival of HAP1 Cells

**Figure 2:**
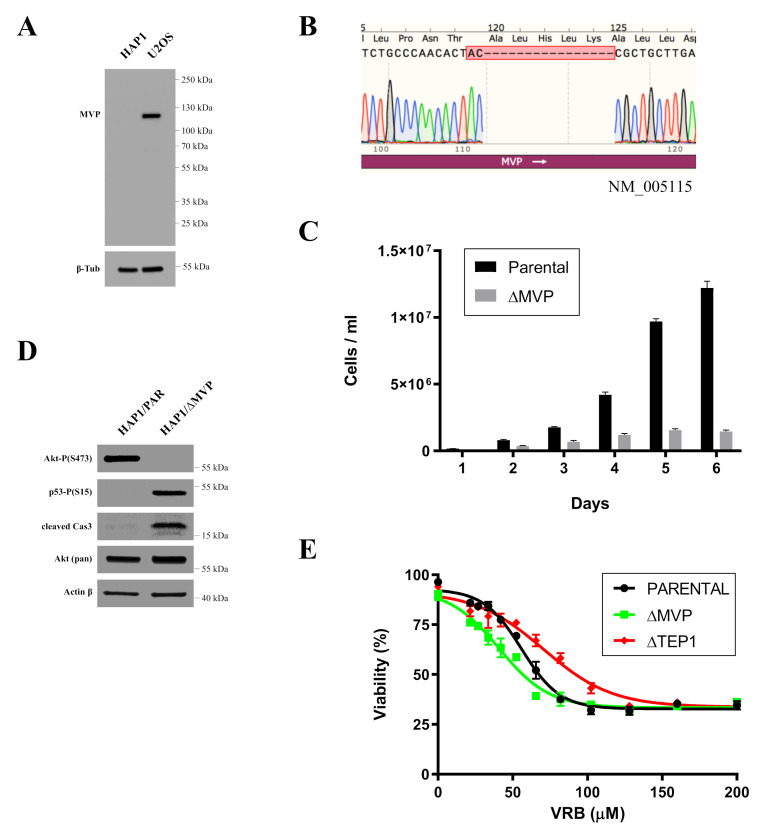
Analysis of MVP-negative HAP1 cells. (**A**) Comparison of endogenous MVP expression in HAP1 and U2OS cells. (**B**) Sequence analysis within the MVP gene in MVP-negative (HAP1-ΔMVP) cells. (**C**) Assessment of the growth of HAP1 and HAP1-ΔMVP parental cells. (**D**) Analysis of proteins related to the processes of proliferation and apoptosis. (**E**) Survival curve of HAP parental cells and MVP- and TEP1-negative cells in the presence of vinorelbine (VRB).

We purchased commercially available unmodified parental HAP1 cells (PAR-HAP1) and CRISPR-Cas9-modified MVP-negative HAP1 cells (ΔMVP-HAP1) to understand MVP’s effect on cell biology. Our first observation was that unmodified, parental HAP1 cells showed undetectable endogenous MVP expression compared to parental U2OS cells (Figure 2A).

Next, we sequenced the MVP gene within the CRISPR-Cas9- modified region as we could not verify the efficiency of modification on the protein level due to low MVP expression in HAP1 cells. In fact, the MVP gene displayed a 16 base pair (bp) deletion when compared to the MVP gene sequence in DNA isolated from PAR-HAP1 cells. This sequence analysis indirectly confirmed that ΔMVP-HAP1 cells were unable to produce the MVP protein (Figure 2B). However, a characteristic feature of ΔMVP-HAP1 cells was their significantly lower viability than unmodified parental cells (PAR-HAP1) (Figure 2C). ΔMVP-HAP1 cells grew very slowly and after a few passages, their ability to divide was completely abolished. This suggests a critical role of MVP in the metabolism of HAP1 cells. In an attempt to answer why ΔMVP-HAP1 cells do not survive, we examined key proteins of selected cell pathways responsible for cell proliferation. We found phosphorylation of a serine 473 (S473) in Akt protein in PAR-HAP1 cells, but not in ΔMVP-HAP1 cells (Figure 2D). Akt plays a key role in the regulation of proliferation and apoptosis. Akt promotes cell survival by phosphorylation and inactivation of several targets including Bad, transcription factors, c-Raf and caspase-9. Lack of S473 phosphorylation is associated with less proliferative activity in HAP1-ΔMVP cells. In addition, we observed phosphorylation of the p53 protein in serine 15, which is usually associated with the DNA damage response and consequently leads to apoptosis [14]. In fact, we observed the presence of an active caspase-3 peptide in MVP-deficient cells (Figure 2D).

We also investigated the effect of the anticancer drug vinorelbine (VRB) on wild-type or ΔMVP HAP1 cells. Previous studies demonstrated a discrepancy in the role of MVP in the development of resistance in cancer cells [12]. Our results indicated that ΔMVP cells were more sensitive to VRB treatment (Figure 2E), which supports the chemopreventive role of MVP. In contrast, TEP1 seems to be neutral or can even stimulate apoptosis in parental cells because its inhibition by the CRISPR-Cas9 method caused higher viability in the presence of VRB when compared to ΔMVP cells (Figure 2E).

### 2.3. Preparation of MVP-Negative U2OS Cells (U2OS-ΔMVP)

**Figure 3:**
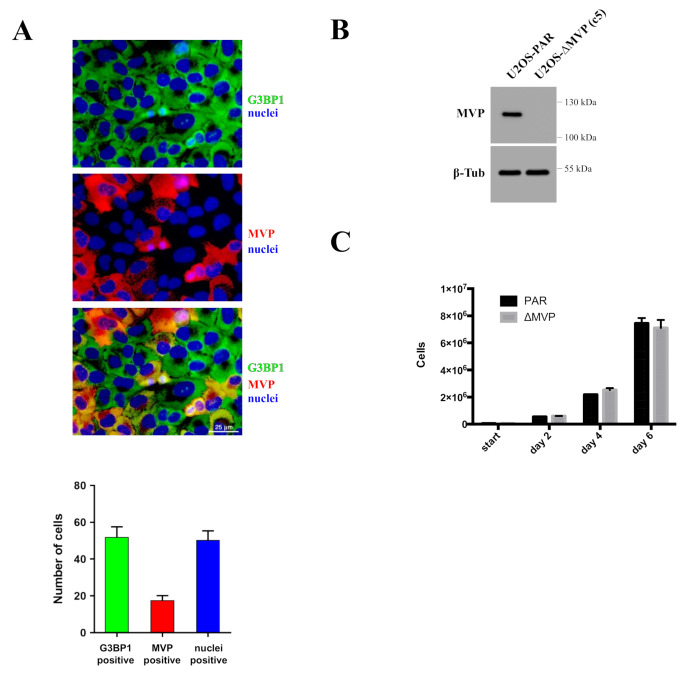
Generation of MVP-negative modified U2OS cells using the CRISPR-Cas9 method. (**A**) Parametric evaluation of the number of cells lacking MVP expression. The G3BP1 protein is expressed in the cytoplasm and was used as a marker for monitoring all cells (green). The MVP is marked red. Cell nuclei are highlighted in blue. The graph shows parametrically the number of total cells (G3BP1—green color, DAPI/nuclei—blue color, MVP—red color). (**B**) Analysis of MVP expression in parental cells (U2OS-PAR) and in modified MVP-negative cells—c5 clone (U2OS-ΔMVP (c5)). (**C**) Cell proliferation analysis on parental (U2OS-PAR) and MVP-negative U2OS cells (U2OS-ΔMVP (c5)).

To test MVP impact on cell survival and proliferation, we ablated MVP in U2OS cells using the CRISPR-Cas9 method. U2OS cells were transfected with two plasmids (the first containing gRNA and Cas9, and the second containing the geneticin resistance gene). Then, we estimated the number of MVP-positive and negative cells based on the immunofluorescence technique (Figure 3A). MVP-negative cells were about 80% of the population. In order to obtain a homogeneous population of MVP-negative U2OS cells, we applied a single cell seeding per one well in a 96-well plate. As a result, we obtained a clonal population that did not express the MVP protein (Figure 3B).

We tested the proliferation of parental U2OS cells and MVP-negative U2OS cells (U2OS-ΔMVP). Surprisingly, in contrast to HAP1-ΔMVP cells, we did not observe any growth abnormalities in U2OS-ΔMVP cells (Figure 3C).

### 2.4. Comparative Analysis of the Transcriptomic Profiles of Parental and MVP-Negative U2OS Cells

**Figure 4:**
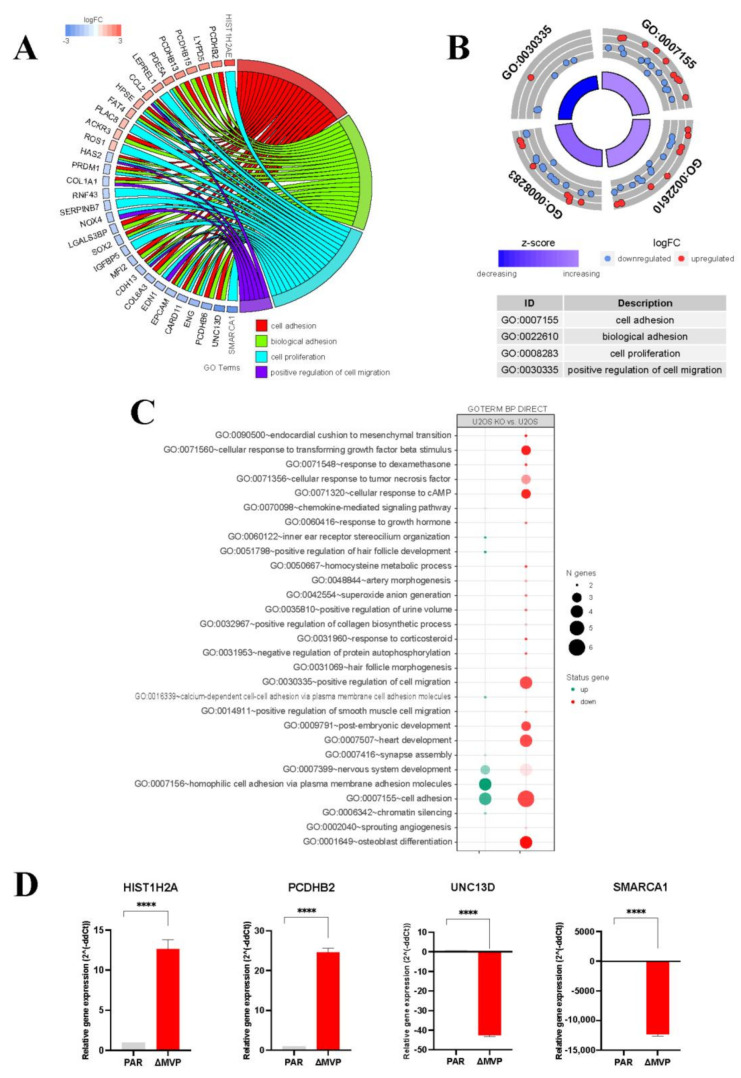
Comparative transcriptomic analysis of parental U2OS cells and MVP-negative U2OS cells (ΔMVP, clone c5). (**A**) Representation of relatively highest and lowest expressed genes in MVP-negative U2OS cells compared to U2OS parental cells. The fluctuations in biological processes in U2OS-ΔMVP cells are marked with colors. (**B**) Bioinformatical analysis of gene ontology (GO) related to biological processes in parental and MVP-negative cells (clone c5). The processes disturbed in MVP-negative U2OS were selected and presented in the table below the pie chart. (**C**) Extended GO analysis shows the contribution of individual processes in parental U2OS cells and MVP-negative U2OS cells. (**D**) RT-qPCR analysis of selected high and low expressing genes in MVP-negative U2OS cells compared to the parental U2OS cells (**** *p* < 0.001).

We used the DNA microarray method developed by Affymetrix in order to study how MVP regulates the biology of U2OS cells. Based on this method, we determined the relative expression level of 34,661 transcripts in the parental U2OS and U2OS-ΔMVP cells. We adopted the following criteria for selecting changes within the comparisons of both tested transcriptomic profiles: expression | fold difference | (absolute value) > 2 and *p* ≤ 0.05. Of the transcripts tested, 48 were elevated in U2OS-ΔMVP cells and 118 were decreased (Figure 4A). This approach allowed for the identification of top high/low expressed genes, both those whose expression is reduced in relation to the parent U2OS cells, and the expression is increased (Figure 4B). Interestingly, all top genes are associated with four biological processes: cell adhesion (GO: 0007155), biological adhesion (GO: 0022610), cell proliferation (GO: 0008283) and positive regulation of cell migration (GO: 0030335) (Figure 4B). The enrichment analysis showed that the expression of genes related to focal adhesion is modified (Figure 4C). To confirm the results obtained with the application of DNA microarrays, we performed the expression analysis of selected genes (HIST1H2A, PCDHB2, UNC13D, SMARCA1) using the qPCR technique (Figure 4D). We confirmed the data obtained from DNA microarrays. As in the data obtained from the microarray, the expression of HIST1H2A and PCDHB2 genes increased in MVP-negative cells, while the expression of the UNC13D and SMARCA1 genes decreased.

### 2.5. Migration Analysis of Parental and MVP-Negative U2OS Cells

**Figure 5:**
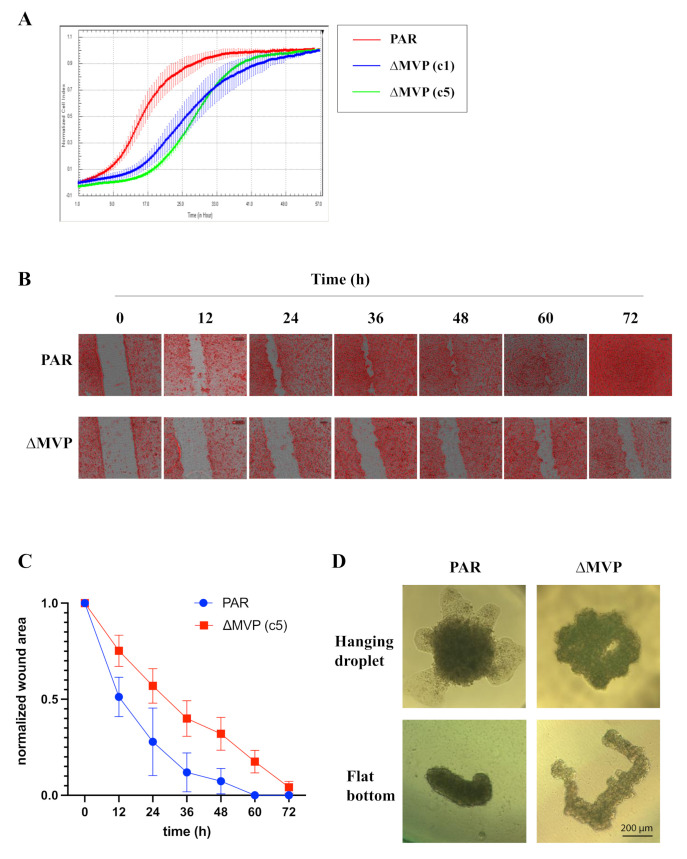
Analysis of migration and morphology of U2OS parental cells and MVP-negative cells (U2OS-ΔMVP). (**A**) Cell migration analysis using the xCELLigance Real-Time Cell Analysis System. Three different cell types were analyzed (parent U2OS, U2OS-ΔMVP, c1 and c5 clones). (**B**) Assessment of cell migration using the wound healing technique of U2OS and U2OS-ΔMVP cells (c5 clone). (**C**) Parametric analysis of the results obtained from cell migration using the wound healing technique. (**D**) Visualization of the morphology of parental U2OS-based and MVP-negative U2OS-based spheroids and using two 3D cultures techniques: hanging drop and flat bottom.

To determine the rate of cell migration, we applied two methods: (1) detection of migrated cells using the xCELLigence technique, which enables us to assess the speed of cell movement in real time, monitored in 15 min intervals, and (2) classic wound healing assay with microscopic analysis. The xCELLigence analysis showed a significant impairment in the migration of ΔMVP cells with two independent clones (Figure 5A). ΔMVP- cells migrated slower than the parental ones. Then, we investigated the migration of cells with wound healing technique [15], and we also observed limited mobility of ΔMVP cells (Figure 5B,C). These data indicate that MVP is strongly responsible for the migratory capacity of cells, which correlated with increased expression in metastatic tumors (Figure 1).

### 2.6. Effect of MVP Expression on Cell Metabolism and Paxillin Phosphorylation

**Figure 6:**
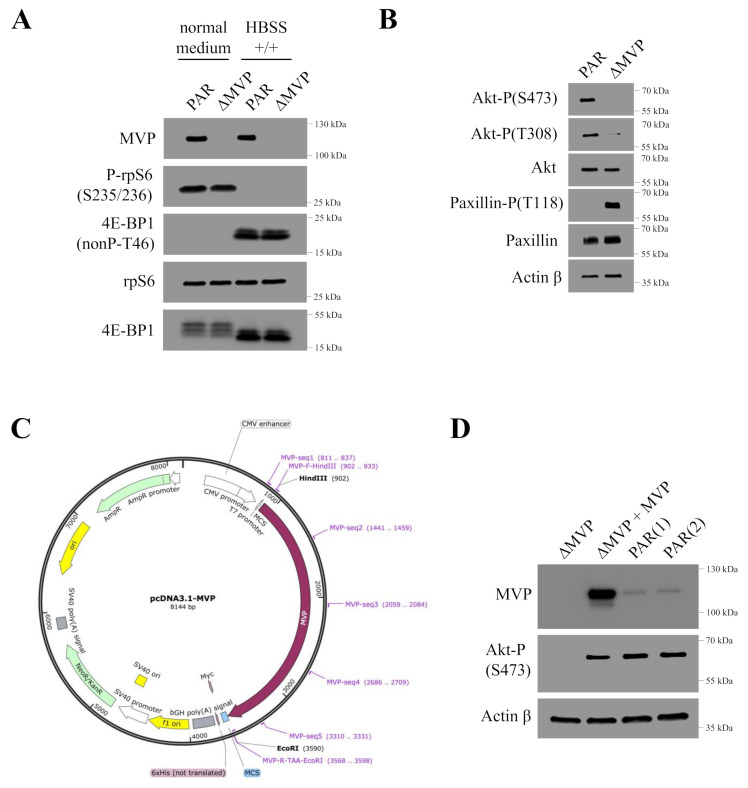
Analysis of PI3K/mTOR/AKT pathway activity in U2OS parental cells and MVP-negative cells (U2OS-ΔMVP, clone c5). (**A**) Analysis of the phosphorylation of proteins related to mTORC1 activity in U2OS parental cells and MVP negative cells (U2OS-ΔMVP). Additionally, starvation conditions were used using HBSS +/+ buffer. (**B**) Analysis of the activity of the Akt protein and paxillin in U2OS and U2OS-ΔMVP cells. (**C**) Map of the expression plasmid containing the sequence of the human MVP. (**D**) Analysis of Akt protein phosphorylation in MVP knockout cells, in cells transfected with the plasmid forming the exogenous MVP, and in U2OS parental cells.

Previous studies indicated the potential role of MVP in regulating the PI3K/mTOR/AKT signaling pathway [13]. We investigated the effect of MVP exclusion on mTOR activity by monitoring the phosphorylation of two key substrate proteins for the mTORC1 enzyme, i.e., the small ribosomal S6 protein and the 4E-BP1 protein regulating the formation of the eIF4F initiation complex [16]; however, we did not observe an effect of MVP reduction on mTOR activity (Figure 6A).

Moreover, our studies based on the microarray technique showed that MVP may participate in the processes related to the cell adhesion (Figure 4B). This was confirmed by migration test and wound healing technique (Figure 5A–C). One of the important subcellular components responsible for cell adhesion and cell–matrix interactions are focal adhesions [17]. Furthermore, they are also crucial in cell migration [18]. In order to verify the effect of MVP on the focal adhesion intradynamics, we checked the paxillin tyrosine 118 (T118) phosphorylation. Previous data suggested that paxillin is phosphorylated at this site by focal adhesion kinase (FAK) [19]. Phospho-paxillin (T118) may provide a docking site for recruitment of other signaling molecules to focal adhesions. It has also been shown that the SH2 domain of Crk binds to the phosphorylated T118 of paxillin [20]. Our results show that paxillin is not phosphorylated in MVP-containing cells but is highly phosphorylated (Tyr118) in MVP-deficient cells (Figure 6B). Paxillin phosphorylation (Tyr118) is not critical to focal adhesion formation, but is characteristic when focal adhesion is formed [21]. Phosphorylation of paxillin (Tyr118) may stabilize focal adhesion and, consequently, limit its dynamics, resulting in lower cell migration capacity.

In order to confirm the participation of MVP in the regulation of the PI3K/mTOR/AKT signaling pathway, we prepared MVP-positive U2OS rescue cells based on the c5 clone (ΔMVP-U2OS) using MVP-coding plasmid (Figure 6C). In ΔMVP U2OS cells, the AKT pathway is blocked, and phosphorylation of Akt (S473) may occur. Expression of exogenous MVP causes a rescue effect and Akt is phosphorylated (S473) (Figure 6D).

## 3. Discussion

Both the MVP protein and whole vaults have been studied for many years, but their real function in the cell has not been fully confirmed. From the beginning, the role of cell vaults in multidrug resistance and to a lesser extent, in the neoplastic process, was suggested [11]. It is not clear enough whether higher or lower expression of MVP supports the process of cancer formation. This is due to the high variability in specific patients. We have previously shown that high level of MVP associates with the tumor grade, while the level of the MVP transcript decreases [22].

In the present study, we focused on assessing the amount of MVP in three types of samples with colorectal cancer obtained from the same patient: normal colon tissue, primary colon cancer and metastasis to the regional lymph nodes. Our studies indicated no significant MVP increase compared to the normal colon tissue and the primary tumor. In contrast, the level of MVP expression increases in the neoplastic metastasis observed in the lymph node (Figure 1). This may indicate the participation of the MVP protein in the epidermal-mesenchymal transition and its participation in promoting the migratory activity of metastatic cells, which was partly previously postulated [13]. In order to investigate the role of MVP protein in the development of neoplastic metastases, we compared the total gene expression in U2OS parental cells and those in which MVP protein expression was inhibited using the CRISPR/Cas9 method. This approach allowed us to determine signaling pathways and processes that have changed (turned off or on) in cells with limited expression of MVP. Our results show that MVP regulates the following processes: cell adhesion, biological adhesion, cell proliferation and positive regulation of cell migration (Figure 5B). These results are directly related to the histopathological features of metastatic cells. 

Indirect confirmation of the role of MVP in the proliferation processes was examined by commercially available HAP1 cells in which the expression of the MVP protein was blocked using the CRISPR/Cas9 method. MVP-negative HAP1 cells (HAP1-ΔMVP) showed a significant impairment in proliferation to the extent that cell division was completely stopped after several passages and the cell population could not be maintained (Figure 2C). It is not clear why HAP1-ΔMVP cells did not survive. However, MVP-negative U2OS cells (U2OS-ΔMVP) did not show significant differences in proliferation rates compared to U2OS parental cells. Perhaps results from HAP1 parental cells had a virtually undetectable amount of MVP compared to parental U2OS cells (Figure 2A). Thus, the inhibition of MVP in HAP1 cells decreased the expression of this protein below a critical level. In contrast, MVP-negative U2OS cells had some residual expression of MVP, which was sufficient for cell survival. In addition, the analysis of the pathways responsible for proliferation and apoptosis showed that proliferation in HAP1-ΔMVP is limited because the AKT pathway is turned off (Figure 2D), and cells enter the process of apoptosis. This is also supported by the observation that the p53 protein underwent phosphorylation, and the active Casp3 peptide appeared in the cell (Figure 2D). Alternatively, it could be possible that U2OS cells have developed mechanisms to mask the absence of MVP.

Using the DNA microarray method, we determined that MVP is involved in the migration processes. We confirmed this phenomenon in subsequent experiments using real-time cell mobility monitoring. MVP-negative cells U2OS migrated slower compared to U2OS parental cells (Figure 5A–C). We extended this study to assess the growth of spheroids (3D cell cultures). The differences in the morphology of the spheroids formed from the parental U2OS cells and from the ΔMVP cells were significant. Cells without MVP formed larger spheroids, indicating that MVP may be involved in cell-to-cell adherance processes. To confirm the role of MVP in these processes, which was initially demonstrated by DNA microarrays, we examined one of the key elements in focal adhesion structure, namely the paxillin protein. Earlier data indicated a direct involvement of paxillin in the formation and functioning of focal adhesions. Paxillin is a multifunctional and multidomain focal adhesion adapting protein that plays an important role as a scaffold in focal adhesion by recruiting structural and signaling molecules involved in cell movement and migration when phosphorylated on specific tyrosine and serine residues. After integrin binds to the extracellular matrix, paxillin is phosphorylated at Tyr31, Tyr118, Ser188, and Ser190, activating numerous signaling cascades that promote cell migration, indicating that regulation of adhesion dynamics is under the control of complex presentation of signaling mechanisms [21]. Our studies indicated that paxillin is phosphorylated at the Tyr118 position (Figure 6B). In addition to growth factors and integrin-dependent adhesion to the extracellular matrix, various stimuli have been shown to induce paxillin phosphorylation [23]. Paxillin is a well-known substrate for the FAK/Src complex, which phosphorylates Tyr31 and Tyr118 in dynamic adhesion, thereby promoting paxillin disassembly from the adhesive complex. In this line, the phosphorylation of Tyr31, Tyr118, Ser188 and Ser190 has been shown to promote migration, suggesting their participation in the adhesion turnover [24]. It seems that Tyr118 hyperphosphorylation observed in MVP-negative cells (Figure 6B) may cause strong stabilization of paxillin in focal adhesion, which results in reduced intradynamics of the latter and lower cell migration capacity.

The MVP protein appears to participate in the regulation of the PI3K/AKT/mTOR pathway, which was previously proposed [13]. Our research indicated that cells lacking the MVP protein showed reduced activity of the AKT pathway (lack of Akt phosphorylation in Ser473 and Tyr 308 residues) (Figure 6B), which directly explains the lower migration capacity of ΔMVP cells. This is also confirmed by the data obtained from the DNA microarrays (Figure 4B), but it does not explain the same proliferative activity of parental and ΔMVP U2OS cells (Figure 3C) in striking contrast to deficient proliferation in ΔMVP HAP1 cells (Figure 2C).

**Figure 7:**
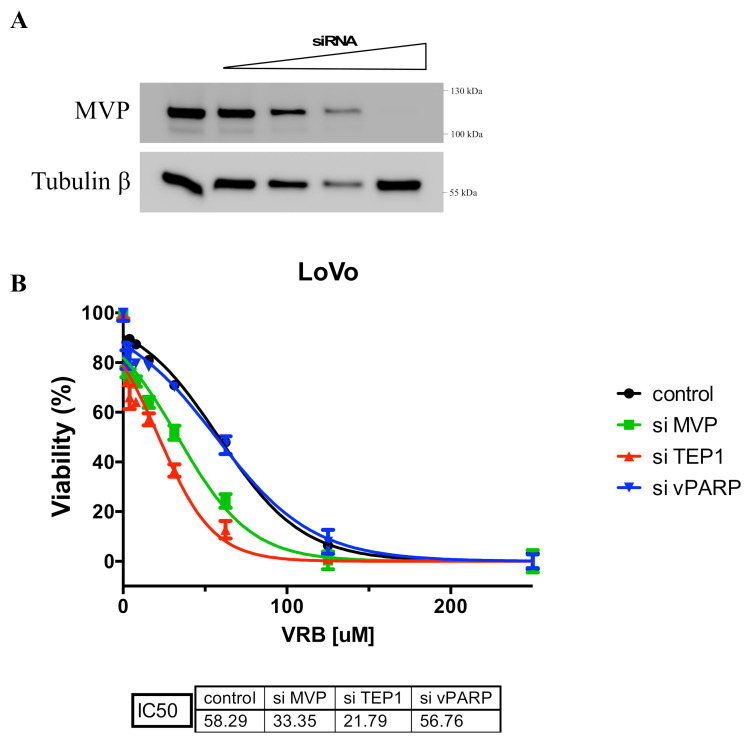
Viability determination for parental U2OS and transiently MVP-, TEP1- and vPARP-negative U2OS in the presence of vinorelbine (VRB). (**A**) Analysis of the effectiveness of MVP silencing by siRNA. (**B**) The survival curves of parental U2OS and transiently MVP-, TEP1- and vPARP-negative U2OS in the presence of vinorelbine (VRB).

Previous studies indicated the role of cellular vault proteins (MVP, TEP1, vPARP) in the resistance of cancer cells to various anticancer drugs [1,2]. We analyzed the role of vault proteins in resistance to VRB. Individual proteins were temporarily silenced by the siRNA method. We then examined cell survival in the presence of VRB. It turned out that inhibition of MVP and TEP1 caused cells to be more sensitive to the cytotoxic effects of VRB (Figure 7). We did not observe this effect when vPARP was transiently disabled. This may indicate the participation of MVP and TEP1 in multidrug resistance, at least for the analyzed VRB. 

We confirmed MVP contribution to the processes of cellular proliferation, migration and metastasis. Together with a previous report, we described that MVP mediates various pathways such as AKT, FAK and ERK. The AKT pathway supports the signal transduction, which promotes survival and growth of cells [25]. Furthermore, AKT phosphorylates many different proteins involved in the cytoskeleton remodeling, which supports the role of MVP in adhesion and metastasis via this pathway [26]. The FAK pathway has a similar effect on the cell growth; however, this pathway also strongly contributes to cell migration [27]. ERK contributes to cell migration, and more importantly, it is responsible for cancerogenesis [28]. Together, this supports MVP as a critical factor in the cancer genesis, proliferation and propagation over the organism.

## 4. Materials and Methods 

### 4.1. Clinical Samples 

Colorectal cancer with matched regional lymph node metastasis carcinoma and adjacent normal colon tissue microarray (TMA) (*n* = 57 cases) (US Biomax, Inc., Maryland, MD, USA; Abcam, Cambridge, MA, USA) were examined. All three samples were collected from the same patient.

### 4.2. Immunohistochemistry

The tissue samples as microsections were dewaxed with xylene, and gradually hydrated. Activity of endogenous peroxidase was blocked by 30 min exposure to 1% H_2_O_2_. Monoclonal mouse anti-human MVP (MVP/LRP) (human) monoclonal antibody (LRP-56) in 1:20 dilution (cat. ALX-801-005-C050, Enzo, USA) were used as the primary antibodies. Tested sections were incubated with primary antibodies overnight at 4 °C, followed by incubation with EnVision Detection System Peroxidase/DAB, Rabbit/Mouse (Dako-Agilent, USA) for 30 min. Every experiment included internal negative controls in which specific antibodies were substituted by sera of a respective species in 0.05 M Tris-HCl, pH~7.6, supplemented with 0.1% bovine albumin (BSA) and 15 mM sodium azide. The sections were then finally reacted with 3,3-diaminobenzidine (DAB), counterstained with hematoxylin, dehydrated and mounted. The protein expression was semiquantitatively evaluated using the Remmele and Stegner immunoreactive score (IRS), taking into account the number of positive cells (PP) and intensity of the color reaction (SI). The final score represented the product of PPxSI and ranged from 0 to 12 points. Additionally, the final value of the PPxSI product ranging between 1 and 2 points characterized a faint, a moderate (3-4) and an intense immunocytochemical (6-12) reaction with individual modifications [29]. Approximately 10 fields of view from each tissue slice were analyzed under Olympus BH-2 light microscope under a 40× objective magnification, and mean scores were calculated. 

### 4.3. Cell Culture 

U2OS cells (human osteosarcoma) were purchased from ATCC (American Type Culture Collection). HAP1-ΔMVP and parental HAP1 were purchased from Horizon Genomics (cat. no. HZGHC003713c002). U2OS cells were grown in Dulbecco’s modified Eagle medium, DMEM (Sigma–Aldrich, St. Louis, MI, USA) with 10% fetal bovine serum (Sigma–Aldrich) and Penicillin-Streptomycin cocktail (Sigma-Aldrich). HAP1 cells were grown in Iscove Modified Dulbecco Media, IMDM (ThermoFisher) with 10% fetal bovine serum (Sigma–Aldrich) and Penicillin-Streptomycin cocktail (Sigma–Aldrich). The cells were grown at 37 °C in 5% CO_2_. 

### 4.4. Cell Viability Assay

Cells were harvested, pelleted and resuspended in 100 µL medium. 10 µL of cells were mixed with 10 µL trypan blue solution (0.4%). Then the viability quantification was measured in Countess II Automated Cell Counter (in Invitrogen™). 

### 4.5. CRISPR-Cas9 Mediated Knockout of MVP 

Oligonucleotides encoding gRNAs targeting the third exon of MVP were designed using CRISPR Design software from the Zhang lab (crispr.mit.edu (accessed on 7 November 2021)). Oligonucleotides were annealed and cloned into pCas-Guide (Origene (Rockville, MD, USA)) according to manufacturer’s protocol. gRNAs target the following sequences within MVP: gRNA(Frw): GATCGAATCAAGCAGCGCCTTTAGAG and gRNA(Rev): AAAACTCTAAAGGCGCTGCTTGATTC. pCas-guide plasmids with cloned with gRNAs were transfected into U2OS cells using Lipofectamine 2000 (Invitrogen). Cells were allowed to recover for seven days and then immunostained for MVP to determine the percentage of knockouts. U2OS cells were first ‘pool cloned’ to enrich knockouts by plating 5–10 cells per well in a 24-well plate. Pool clones were screened by immunofluorescence. Pool cloned U2OS cells and original transfection of U2OS were cloned by limiting dilution and screened by immunofluorescence and Western blotting.

### 4.6. Microarray Expression Study 

The microarray study was performed as described in detail elsewhere [30]. The total RNA isolated from two types of cells was pooled into four samples per group (parental U2OS and ΔMVP-U2OS). The protocol including in vitro transcription, biotin labeling, and cDNA fragmentation was performed using the Affymetrix GeneChip IVT express kit (Affymetrix, Santa Clara, CA, USA). Then, the biotin labeled cDNA were hybridized with the Affymetrix Gene Chip Human Genome U219 microarrays together with appropriate internal controls. The hybridization was performed in the AccuBlockTM digital dry bath hybridization oven (Labnet International, Inc., Edison, NJ, USA) at 45 °C for 16 h. Subsequently, the microarrays were washed and stained by means of the Affymetrix GeneAtlas Fluidics Station (Affymetrix, Santa Clara, CA, USA). The microarrays were scanned using the imaging station of the GeneAtlas System. Initial analysis of the scanned microarrays was carried out with Affymetrix GeneAtlas TM Operating Software.

### 4.7. Microarray Data Analysis 

The generated CEL files were subjected to further analysis using the R statistical language and bioconductor package with the relevant bioconductor libraries. The robust multiarray average (RMA) normalization algorithm implemented in the “Affy” library was used for normalization, background correction, and calculation of the expression values of all of the examined genes [31]. Assigned biological annotations were accessed from “pd.hugene.2.1.st” library that was used for the mapping of normalized gene expression values with their symbols, gene names, and Entrez IDs, leading to a complete gene data table. Differential expression and statistical assessment were determined by applying the linear models for microarray data implemented in the “limma” library [32]. The accepted cut-off criteria were based on both differences in expression fold change (FC) greater than abs. 1.5 and 10% false discovery rate (FDR) correction. Genes that fulfilled the selection criteria were considered as differentially expressed genes. 

### 4.8. Assignment of Differentially Expressed Genes to Relevant Gene Ontology (GO) Terms

All differentially expressed genes from both comparisons (parental U2OS and ΔMVP-U2OS) were subjected to functional annotation and clusterization using the DAVID (Database for Annotation, Visualization, and Integrated Discovery) bioinformatics tools. Gene symbols of differentially expressed genes were uploaded to DAVID by the “RDAVIDWebService” bioconductor library, where DEGs were assigned to relevant GO terms, with subsequent selection of significantly enriched GO terms. The *p*-values of selected GO terms were corrected using Benjamini–Hochberg false discovery rate method described as adjusted *p*-values. GO groups essential for adrenal physiology with adjusted *p*-values below 0.05 were visualized using bubble plots. Details of the genes belonging to particular GO terms with their fold change values were presented as circos plots using “GOplot” library [33].

### 4.9. Total RNA Isolation 

Total RNA was isolated from cultured cells using Universal RNA Purificatin Kit (EURx, Gdańsk, Poland) according to manufacturer’s instruction. The isolated total RNA was quantified using NanoDrop (ThermoScientific, Waltham, MA, USA) and its integrity was checked on 1% agarose gel.

### 4.10. RT-qPCR 

The reaction was performed using Luna Universal qPCR Master mix (New England Biolabs, Ipswich, MA, USA). Previously cDNA was synthesized using LunaScript RT SuperMix kit. The primers for qPCR were purchased from Bio-Rad (HPRT, A2M, GAPDH, PCDH, H2AE, SMARCA1, UNC13D). All reactions were carried out in triplicate.

### 4.11. xCELLigance (RTCA Cell Migration Assay)

To verify the effect of MVP on the migration rate of U2OS cells (PAR-U2OS and U2OS-ΔMVP) together with the identification of the intracellular mechanism, we applied an electrical impedance-based cell proliferation assay, named the real-time cell Analyzer (RTCA, Roche Applied Science, GmbH, Penzberg, Germany). The RTCA system detects fluctuations in electrical impedance on the integrated sensory electrodes located at the bottom of the chamber’s 16-hole slide plates (E-Plate 16), which are covered by dividing cells. The down chamber is fulfilled with BSA (bovine serum albumins), as an attractant. Cells migrate from the upper chamber to the bottom one where they change the impedance on the surface. Electrical impedance is measured at 15 min intervals throughout the cultivation period. The main RTCA readout is the “cell migration index”—a measurable parameter corresponding to the relative change in electrical impedance depending on the rate of migration in the cultivated cells. All experiments were carried out in triplicate.

### 4.12. Wound Healing

U2OS parental cells and U2OS-ΔMVP were grown in the 4-well plate till full confluency. The scratch was carried out using 200 µL pipette yellow tip. Then the wound healing was measured in time. Finally, the migration rate was calculated using ImageJ. All measures were carried out at least in triplicate. 

### 4.13. 3D Cells Cultures

Cells were grown using two different methods of 3D culturing: hanging droplet and flat bottom. 10*^3^* cells were seeded and grown for 5 days. Then, the size and morphology were analyzed using a light inverted microscope (Zeiss, Jena, Germany). All measures were carried out in at least triplicate.

### 4.14. siRNA Transfection

105 U2OS cells were seeded in the 6-well plates and grown for 24 h. Then, the first transfection was carried out with the following substrates: 100 pmol siRNA (Thermo Scientific, Dharmacon, all are SmartPools), 2.5 μL Lipofectamine 2000 (Invitrogen) in OPTI-MEM (Life Technologies, Carlsbad, CA, USA) and cells were incubated with siRNA for 24 h. Between the first and second transfection cells were cultured in DMEM with 10% fetal bovine serum for 24 h. The second transfection was carried out with the same conditions as the first. Finally, cells were collected and counted.

### 4.15. Western Blotting

Cells were grown to 80% confluence in 6-weel plates. Then cells were washed with HBSS-/- buffer (Gibco) and total protein isolation was performed using Minute™ Detergent-Free protein extraction kit for animal tissues and cultured cells (Invent Biotechnologies, Plymouth, MN, USA) according to manufacturer’s instruction. Then, the total proteins were quantified using Pierce BCA protein assay (Thermo Scientific). The equal amount of proteins were applied on 4–20% TGX gel (BioRad, Hercules, CA, USA). The blotting was carried out using Turbo-blot system (BioRad). The membrane was incubated with non-fat milk (5%) for 1h. Then, primary antibodies were applied and incubated overnight at 4 °C. The second antibodies were applied for 1h. Finally, proteins were detected using SuperSignal™ West Pico PLUS chemiluminescent substrate (Thermo Scientific) and the images were processed using Amersham Imager 600 system. 

### 4.16. Antibodies 

Antibodies were purchased from Cell Signaling Technologies (USA): β-Tubulin (number 2128), rpS6-P(S235/236) (number 2211), 4E-BP1-nonP(T46) (number 4923), 4E-BP1 (number 9644), Akt-P(S473) (number 4060), Akt-P(T308) (number 4056), Akt (number 4685), Paxillin-P(T118) (number 69363), Paxillin (number 12065), and Actin (number 4970). Monoclonal mouse anti-human MVP (LRP-56) antibodies were purchased from Enzo (cat. no. ALX-801-005-C050, Enzo). 

### 4.17. Plasmid

MVP-expressing plasmid pCMV6-XL5 were purchased from Origene (SC114118).

### 4.18. Statistical Analysis

Unpaired Student’s *t*-test was used in all performed experiments. *p*-value was calculated using GraphPad (Prism) software. All experiments were carried out at least in triplicate.

## Data Availability

Not applicable.
